# Associations of dietary phosphorus-protein ratio, phosphorus-energy ratio, and protein-energy ratio with mortality in peritoneal dialysis patients: a retrospective cohort study

**DOI:** 10.3389/fnut.2026.1798804

**Published:** 2026-06-09

**Authors:** Zi-Zhen Wang, Ke Xu, Su-Xuan Liu, Si-Yu Yang, Xin-Xin Chen, Chun-Yan Su, Wen Tang

**Affiliations:** Department of Nephrology, Peking University Third Hospital, Beijing, China

**Keywords:** cohort study, dietary intake, energy, peritoneal dialysis, phosphorus, protein

## Abstract

**Background:**

Optimal nutritional management in patients undergoing peritoneal dialysis (PD) requires balancing phosphorus restriction with adequate protein and energy intake. Nutrient density-based indicators, including the phosphorus-protein ratio, phosphorus-energy ratio, and protein-energy ratio, may better capture dietary structure than absolute nutrient intake; however, their prognostic significance in PD patients remains unclear.

**Method:**

This retrospective cohort study included adult patients who initiated PD at a single center between January 2006 and June 2021 and were followed until June 2023. Dietary phosphorus, protein, and energy intake were assessed using 3-day dietary records collected six months after PD initiation. The phosphorus-protein ratio, phosphorus-energy ratio, and protein-energy ratio were calculated as exposure indicators. Restricted cubic spline models were used to explore potential nonlinear associations with all-cause and cardiovascular mortality. Associations with all-cause mortality were evaluated using Cox proportional hazards models, while cardiovascular mortality was analyzed using both Cox and Fine-Gray competing risk models. In addition, sensitivity analyses were performed using methods such as excluding early deaths and incorporating repeated measurement data.

**Results:**

A total of 794 PD patients were included, with a median follow-up of 47 months, during which 412 deaths occurred, including 158 cardiovascular deaths. In the fully adjusted model, the phosphorus-protein ratio showed a significant nonlinear association with all-cause mortality (*p* for nonlinearity = 0.027), while the nonlinear association with cardiovascular mortality was borderline significant (*p* = 0.081). Compared with the second quintile [14.41 (14.15–14.62)mg/g], higher quintiles were associated with higher cardiovascular mortality. These findings were consistent in competing risk analyses. In contrast, the phosphorus-energy ratio (mg/100 kcal, HR = 1.015, 95% CI 1.004–1.025, *p* = 0.006), and protein-energy ratio (%, HR = 1.050, 95% CI 1.011–1.091, *p* = 0.011) showed linear positive associations with all-cause mortality.

**Conclusion:**

Among patients undergoing PD, baseline dietary nutrient density indicators exhibit distinct associations with mortality outcomes. These findings raise the possibility that dietary structure, beyond absolute intake of individual nutrients, might offer additional insights for risk stratification and nutritional management in PD patients.

## Introduction

The long-term prognosis of patients undergoing peritoneal dialysis (PD), particularly the persistently high burden of cardiovascular mortality, remains a major clinical challenge. According to the United States Renal Data System (USRDS) 2025 Annual Data Report (1), cardiovascular (heart-related) conditions represent the leading cause of death among patients receiving PD, accounting for more than 39.8% of all deaths. Moreover, mortality among older individuals undergoing dialysis (including both hemodialysis and PD) with Medicare fee-for-service coverage was substantially higher than that observed in patients with major chronic conditions such as cancer, diabetes, heart failure, stroke, or acute myocardial infarction. Among the multiple determinants of prognosis in PD patients, nutritional status has consistently been identified as a key factor associated with survival (2, 3). Accordingly, the KDIGO guidelines emphasize that nutritional management constitutes an integral component of comprehensive chronic kidney disease (CKD) care and should be delivered by trained kidney nutrition professionals within an individualized patient-centered framework (4).

In the nutritional management of PD patients, maintaining an appropriate balance among phosphorus, protein, and energy intake is crucial. Hyperphosphatemia has been widely recognized as a potent contributor to vascular calcification. Experimental and clinical evidence indicate that excess phosphorus can directly promote the phenotypic transformation of vascular smooth muscle cells into osteogenic-like cells, facilitating the deposition of hydroxyapatite crystals within the vascular wall and thereby contributing to vascular stiffening and atherosclerotic processes (5). Conversely, insufficient protein intake has been associated with protein-energy wasting (PEW) and adverse clinical outcomes in patients undergoing PD (6, 7). PEW is defined as a state characterized by the depletion of body protein and energy reserves, including reductions in both lean body mass and fat mass (8). Previous studies have reported that the prevalence of PEW among dialysis patients ranges from 28 to 54%, and that PEW is independently associated with increased mortality and impaired quality of life (8, 9). Furthermore, inadequate energy intake necessitates increased endogenous protein catabolism to meet metabolic demands, thereby accelerating the development and progression of PEW (10). However, in mixed diets, particularly those rich in animal-derived foods, organic phosphorus is predominantly protein-bound, resulting in a strong positive correlation between dietary phosphorus intake and protein intake (11, 12). This interdependence creates a fundamental nutritional dilemma for PD patients. Consequently, in routine clinical practice, dietary strategies that consider individual nutrients in isolation, such as restricting phosphorus intake without accounting for protein or energy adequacy, may lead to conflicting recommendations and suboptimal nutritional management.

To address this challenge, researchers have proposed the use of nutrient density-based indicators, such as the phosphorus-protein ratio, phosphorus-energy ratio, and protein-energy ratio, to better characterize the nutritional composition and dietary quality of foods (13–15). Although these indices are increasingly being incorporated into dietary counseling, most existing research has focused on hemodialysis populations. Evidence regarding the prognostic significance of these nutrient density measures in PD patients remains scarce. Therefore, this retrospective cohort study was designed to investigate the associations of dietary phosphorus-protein ratio, phosphorus-energy ratio, and protein-energy ratio with prognostic outcomes in PD patients, aiming to provide a stronger evidence base for nutritional management in PD.

## Methods

### Study design and population

This is a single-center retrospective cohort study. The patients who started PD at Peking University Third Hospital between January 2006 and June 2021 were included in this study. Inclusion criteria were as follows: (1) duration of PD exceeding 6 months; (2) aged 18 years and above. Patients with multiple organ failure or malignant tumors were excluded. All subjects were followed up until death, transfer to hemodialysis, kidney transplantation, or the end of the study (June 2023). This study has been approved by the Medical Ethics Committee of Peking University Third Hospital (approval number: IRB 2024–096-03). As this study is a retrospective, non-interventional design and utilizes a de-identified dataset, individual patient informed consent forms were not obtained.

The participants’ demographic data, medical history, and blood chemistry at the sixth month of dialysis were collected.

### Exposure ascertainment

Dietary phosphorus, protein, and energy intake were calculated from three-day dietary records (including two working days and one weekend). The first batch of dietary records collected in the sixth month after the start of dialysis served as baseline dietary data. During the follow-up, all patients visited the PD clinic and received dietary assessment regularly.

After verification by a trained renal dietitian using food models, the information from the food diary was entered into a computer software program (PD Information Management System of Peking University Third Hospital), and the corresponding dietary intake was calculated. The nutrient source database was derived from the China Food Composition Table (16). For each assessment, the mean daily intake of total phosphorus (mg/day), protein (g/day), and energy (kcal/day) was calculated. The phosphorus-protein ratio (mg/g) was derived by dividing total dietary phosphorus by total dietary protein. The phosphorus-energy ratio was defined as total dietary phosphorus intake divided by total energy intake and multiplied by 100. The protein-energy ratio (PER) was defined as the percentage of total energy obtained from protein (17). PER was calculated using the equation:


$$\mathrm{PER}(\%)=\frac{\text{Protein intake}(g)\times 4\text{kcal}/g}{\text{Total energy intake}(\text{kcal})}\times 100\%$$

For the present analysis, the baseline phosphorus-protein ratio, phosphorus-energy ratio, and PER were used as the primary exposure.

### Outcome ascertainment

The primary outcome was all-cause mortality. The secondary outcome was cardiovascular mortality, with cardiovascular death defined as fatal events resulting from myocardial infarction, sudden cardiac death, heart failure, aortic aneurysm rupture, cerebrovascular events, or other cardiovascular etiologies. For analyses of cardiovascular mortality, non-cardiovascular death, kidney transplantation, renal function recovery, and loss to follow-up were treated as competing events in the Fine-Gray sub-distribution hazard model.

### Statistical methods

To investigate potential linear or nonlinear associations between the phosphorus-protein ratio, phosphorus-energy ratio, PER, and mortality risk, all exposure variables were modeled as continuous variables using restricted cubic spline (RCS) functions. The number of knots in RCS models is typically set between 3 and 8 (18). For many datasets, four knots provide an optimal balance between model fit, curve smoothness, and avoidance of overfitting (19); therefore, four knots were prespecified for primary analyses; however, model fit was further explored using alternative knot numbers.

Based on the exploratory RCS analyses, Cox proportional hazards regression models were subsequently applied to assess the associations between baseline exposures and mortality outcomes, with hazard ratios (HRs) and 95% confidence intervals (CIs) estimated. The proportional hazards assumption was evaluated using Schoenfeld residuals. According to prior evidence, clinical considerations, and results from univariable analyses, three models were constructed:

Model 1 (Unadjusted model): no covariate adjustment.
Model 2 (Case-mix model): adjusted for age, sex, diabetes mellitus, and history of cardiovascular or cerebrovascular disease (CCVD).
Model 3 (Expanded case-mix model): additionally adjusted for serum urea, serum albumin, and daily energy intake, daily protein intake, or daily phosphorus intake, as appropriate. To avoid multicollinearity, the dietary component mathematically embedded in each exposure indicator was excluded from the corresponding model. Specifically, daily energy intake was adjusted for when analyzing the phosphorus-protein ratio; daily protein intake was adjusted for when analyzing the phosphorus-energy ratio; and daily phosphorus intake was adjusted for when analyzing PER. The fully adjusted Cox models for all-cause mortality and cardiovascular mortality meet the proportional hazards assumption, as confirmed by the corresponding tests (Supplementary Table S1).

In the primary analyses, considering that competing events such as malignancy and multiple organ failure might occur during follow-up and compete with cardiovascular death, Fine-Gray competing risk models were applied as sensitivity analyses for cardiovascular mortality. Competing events were incorporated into the models, and subdistribution hazard ratios (sHRs) with 95% CIs were calculated to assess the robustness of the association between the phosphorus-protein ratio and cardiovascular mortality. Stratified analyses were conducted according to age (≤60 vs. >60 years), sex, diabetes mellitus status, and history of CCVD to evaluate the consistency of associations across subgroups. Interaction terms were introduced into the models to formally test for effect modification. Additionally, we conducted sensitivity analyses excluding early deaths. To reduce the potential impact of reverse causation (i.e., patients with severe disease altering their dietary patterns due to deteriorating health at the early stage of follow-up), we first excluded participants with a follow-up time less than 1 year and repeated the primary Cox proportional hazards models in the remaining cohort. Furthermore, we calculated the average intake of dietary phosphorus, energy, and protein based on repeated measurements during the first 2 years of follow-up to better reflect long-term dietary exposure. Participants who died within the first 2 years were also excluded, and the primary Cox proportional hazards models were repeated in this restricted cohort.

All statistical analyses were performed using R 4.5.0 software. A two-sided *p <* 0.05 was considered statistically significant.

## Results

### Participants’ characteristics

A total of 794 patients were included in the study. The median age was 61.09 years, 53.4% were male, and the median BMI was 23.09 kg/m^2^. Overall, 43.2% of patients had diabetes, 88.9% had hypertension, and 27.5% had a history of cardiovascular or cerebrovascular disease. Table 1 presents the baseline characteristics of enrolled participants in this study. During a median follow-up of 47 months, a total of 412 deaths occurred, of which 158 were attributed to cardiovascular causes. Univariable Cox regression analyses of baseline variables are shown in Supplementary Table S2.

**Table 1:** Baseline characteristics of 794 peritoneal dialysis patients.

Variables	Data
Patients characteristics	
Age (years)	61.09 (48.01, 71.84)
Sex (males, %)	424.00 (53.40)
BMI (kg/m^2^)	23.09 (21.08, 25.51)
Diabetes (%)	343.00 (43.20)
Hypertension (%)	706.00 (88.90)
History of CCVD (%)	218.00 (27.50)
Laboratory parameters	
Kt/V	1.98 (1.63, 2.42)
GFR	2.45 (1.12, 3.99)
Serum urea (mmol/L)	19.80 (16.22, 23.78)
Serum creatinine (mg/dL)	7.78 (6.21, 9.67)
Serum albumin (g/L)	37.40 (34.30, 40.00)
Serum phosphorus (mmol/L)	1.48 (1.24, 1.73)
Nutrition parameters	
Phosphorus intake (mg/day)	759.86 (602.39, 884.94)
Protein intake (g/day)	49.44 (39.48, 58.32)
nDPI (g/kg/day)	0.83 (0.68, 1.00)
DEI (kcal/day)	1436.45 (1186.46, 1731.33)
nDEI (kcal/kg/day)	24.84 (20.92, 29.05)
Phosphorus-protein ratio (mg/g)	15.30 (14.14, 16.58)
Phosphorus-energy ratio (mg/100 kcal)	51.70 (45.10, 58.17)
PER (%)	13.44 (11.86, 15.16)

Continuous variables are presented as median (Q1, Q3); categorical variables are presented as number (percentage). BMI, body mass index; CCVD, cardiovascular or cerebrovascular disease; GFR, glomerular filtration rate; nDPI, normalized dietary protein intake; DEI, dietary energy intake; nDEI, normalized dietary energy intake; PER, protein-energy ratio.

### Association of phosphorus-protein ratio and mortality

**Figure 1:**
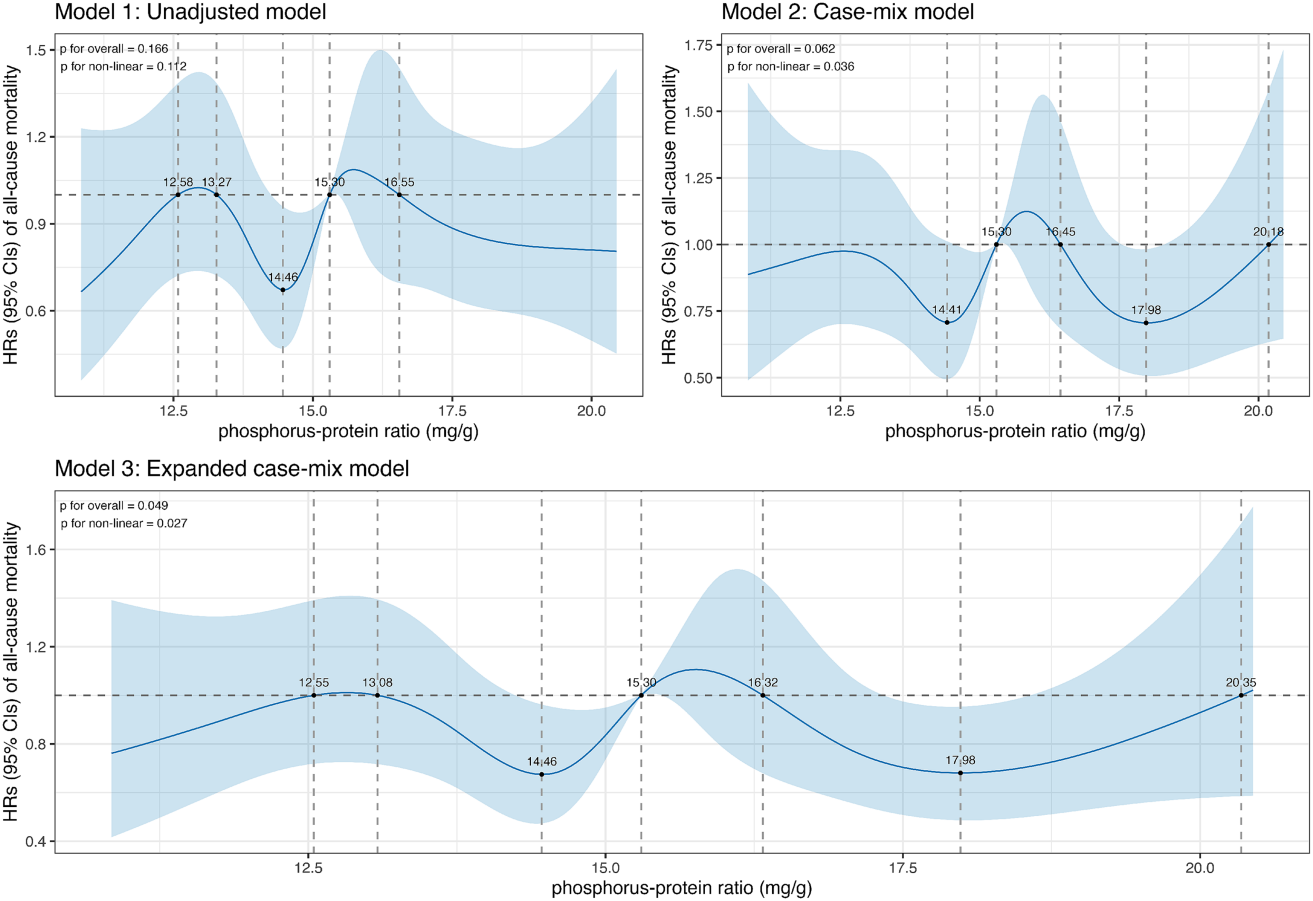
Restricted cubic spline curves showing the association between the phosphorus-protein ratio and all-cause mortality. Solid lines represent the estimated hazard ratios from the restricted cubic spline model, and shaded bands represent the 95% confidence intervals. The horizontal dashed line indicates a hazard ratio of 1.0. (Model 1: unadjusted. Model 2: adjusted for sex, age, diabetes and history of cardiovascular or cerebrovascular disease. Model 3: additionally adjusted for serum urea, serum albumin and daily energy intake).

**Figure 2:**
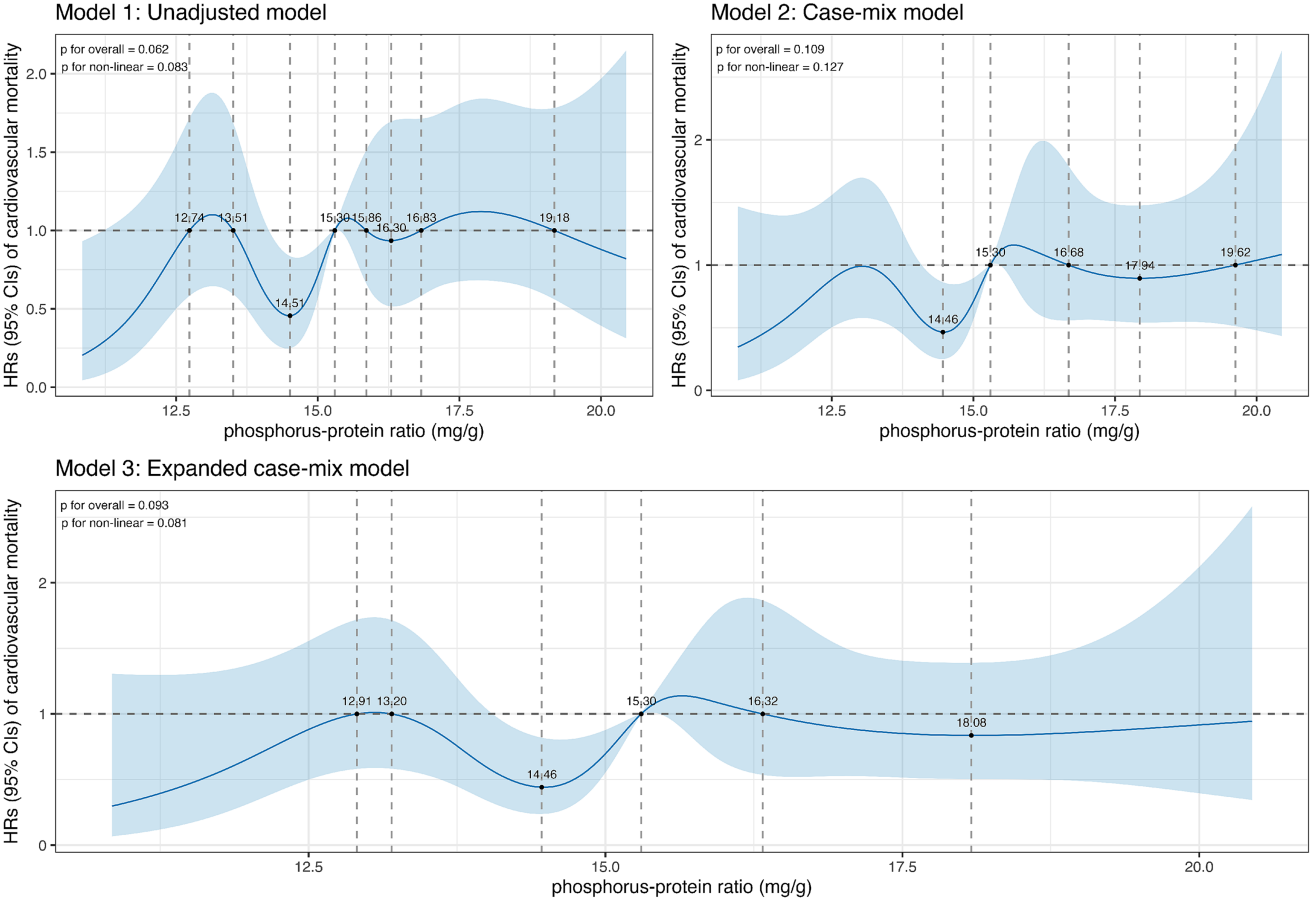
Restricted cubic spline curves showing the association between phosphorus-protein ratio and cardiovascular mortality. Solid lines represent the estimated hazard ratios from the restricted cubic spline model, and shaded bands represent the 95% confidence intervals. The horizontal dashed line indicates a hazard ratio of 1.0. (Model 1: unadjusted; model 2: adjusted for sex, age, diabetes, and history of cardiovascular or cerebrovascular disease; model 3: additionally adjusted for serum urea, serum albumin, and daily energy intake).

In the four-knot RCS analyses, both the *p* value for overall association and the p value for nonlinearity exceeded 0.05. Following Harrell’s recommendation (19), the Akaike Information Criterion (AIC) was used to compare model fit across RCS models with different numbers of knots. Accordingly, RCS models with 3 and 5–8 knots were constructed, and the seven-knot model demonstrated the best fit (Supplementary Table S3). In the expanded seven-knot RCS model, the phosphorus-protein ratio exhibited a significant nonlinear association with all-cause mortality (*p* for overall = 0.049, *p* for nonlinear = 0.027, Figure 1) and a borderline significant nonlinear association with cardiovascular mortality (*p* for overall = 0.093, *p* for nonlinear = 0.081, Figure 2).

Based on these findings, the phosphorus-protein ratio was categorized into quintiles (Q1-Q5) for Cox regression analyses. RCS analysis suggested a nonlinear association between the phosphorus-protein ratio and mortality. Kaplan–Meier survival curves further showed that participants in the Q2 group had the highest survival probability among the quintiles (Supplementary Figures S1, S2), indicating that this range may correspond to a relatively lower-risk region rather than other exposure levels. Therefore, Q2 was selected as the reference category in the categorical analyses. As shown in Table 2, in the expanded model, compared with Q2, the risks of cardiovascular mortality were significantly higher in Q3 (HR = 2.208, 95%CI 1.279–3.810, *p* = 0.004) and Q4 (HR = 2.132, 95%CI 1.215–3.741, *p* = 0.008). In addition, Q4 was associated with an increased risk of all-cause mortality (HR = 1.374, 95%CI 1.009–1.871, *p* = 0.044). In the fully adjusted model, the RCS analysis indicated that the risk of both all-cause and cardiovascular mortality was lowest at a phosphorus-protein ratio of 14.46 mg/g. When the phosphorus-protein ratio exceeded 15.3 mg/g, the risk of mortality began to increase.

**Table 2:** Associations of phosphorus-protein ratio quintiles with cardiovascular mortality and all-cause mortality.

Quintile	Median [IQR]	Model1		Model2		Model3	
		HR [95%CI]	*p* value	HR[95%CI]	*p* value	HR [95%CI]	*p* value
Cardiovascular mortality							
Q1	12.95[12.14,13.46]	1.734 [0.995–3.022]	0.052	1.637 [0.938–2.857]	0.083	1.740 [0.997–3.039]	0.051
Q2	14.41[14.15,14.62]	reference		reference		reference	
Q3	15.30[15.10,15.50]	2.097 [1.220–3.605]	0.007	1.997 [1.160–3.441]	0.013	2.208 [1.279–3.810]	0.004
Q4	16.21[15.91,16.58]	2.037 [1.168–3.551]	0.012	2.162 [1.234–3.789]	0.007	2.132 [1.215–3.741]	0.008
Q5	17.91[17.38,18.62]	1.950 [1.122–3.390]	0.018	1.653 [0.947–2.884]	0.077	1.646 [0.942–2.879]	0.080
All-cause mortality							
Q1	12.95[12.14,13.46]	1.187 [0.872–1.615]	0.276	1.155 [0.848–1.573]	0.362	1.198 [0.878–1.633]	0.254
Q2	14.41[14.15,14.62]	reference		reference		reference	
Q3	15.30[15.10,15.50]	1.275 [0.937–1.735]	0.122	1.224 [0.898–1.668]	0.201	1.324 [0.971–1.804]	0.076
Q4	16.21[15.91,16.58]	1.468 [1.081–1.993]	0.014	1.408 [1.035–1.915]	0.029	1.374 [1.009–1.871]	0.044
Q5	17.91[17.38,18.62]	1.093 [0.792–1.510]	0.588	0.939 [0.677–1.301]	0.704	0.946 [0.681–1.312]	0.738

Model 1: unadjusted. Model 2: adjusted for sex, age, diabetes and history of cardiovascular or cerebrovascular disease. Model 3: additionally adjusted for serum urea, serum albumin and daily energy intake.

### Association of phosphorus-energy ratio and mortality

**Figure 3:**
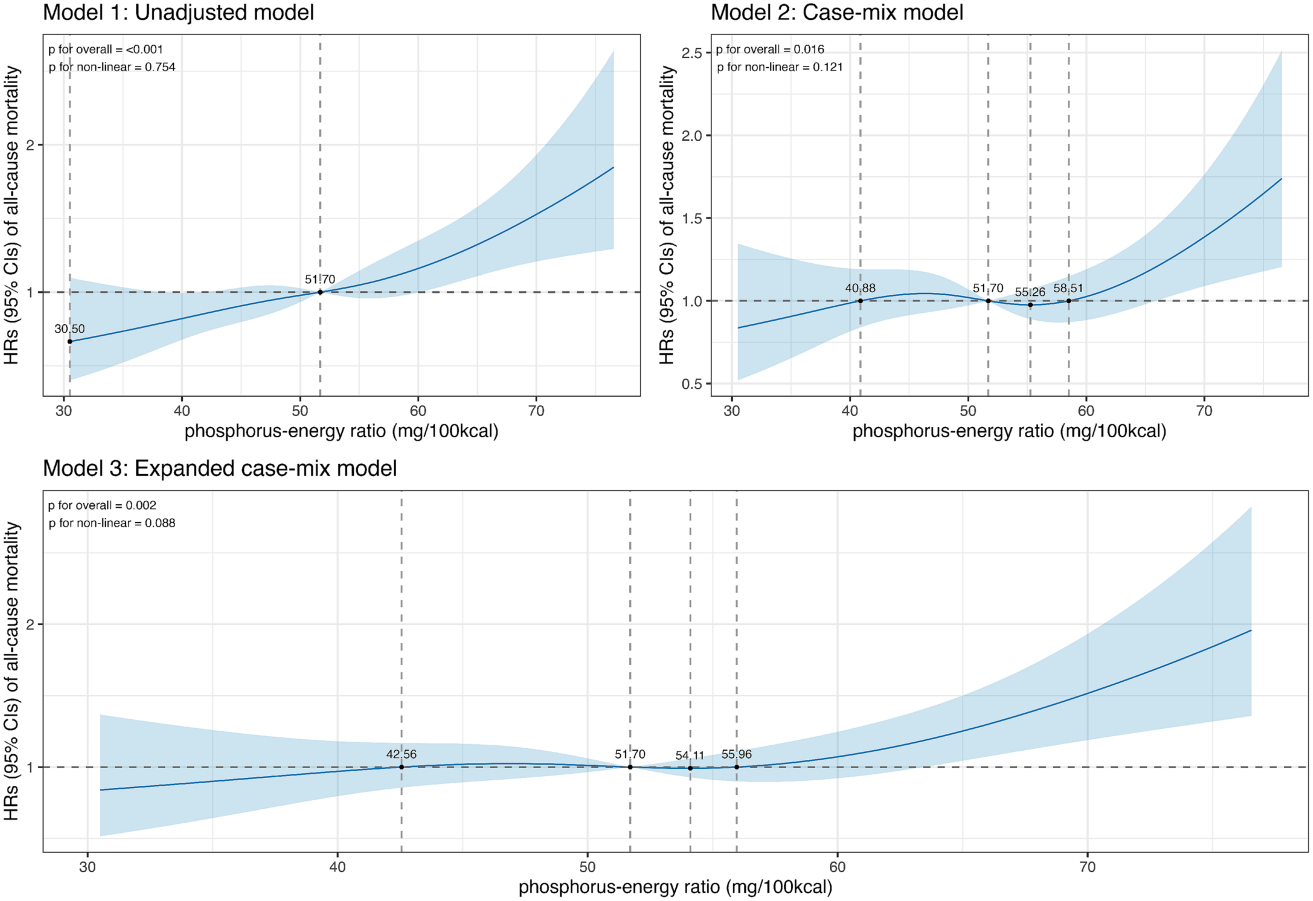
Restricted cubic spline analyses of the association between phosphorus-energy ratio and all-cause mortality. Solid lines represent the estimated hazard ratios from the restricted cubic spline model, and shaded bands represent the 95% confidence intervals. The horizontal dashed line indicates a hazard ratio of 1.0. (Model 1: unadjusted. Model 2: adjusted for sex, age, diabetes, and history of cardiovascular or cerebrovascular disease. Model 3: additionally adjusted for serum urea, serum albumin, and daily protein intake).

In the four-knot RCS analyses, the phosphorus-energy ratio showed a linear association with all-cause mortality across all three models (*p* for overall < 0.05, *p* for nonlinear > 0.05, Figure 3). No significant linear or nonlinear association was observed with cardiovascular mortality (*p* for overall > 0.05, *p* for nonlinear > 0.05, Supplementary Figure S3).

Accordingly, the phosphorus-energy ratio was treated as a continuous variable in Cox regression analyses. Across all models, higher phosphorus-energy ratios were associated with increased mortality risk. In the expanded model, each 1 mg/100 kcal increase in the phosphorus-energy ratio was associated with a 1.015-fold increase in the risk of all-cause mortality (HR = 1.015, 95% CI 1.004–1.025, *p* = 0.006, Table 3). Consistent with the RCS results, Cox regression analyses showed no significant association between the phosphorus-energy ratio and cardiovascular mortality in the expanded model (Supplementary Table S4).

**Table 3:** Associations of phosphorus-energy ratio and protein-energy ratio (PER) with all-cause mortality.

Variables	Model1		Model2		Model3	
	HR [95%CI]	*p* value	HR[95%CI]	*p* value	HR [95%CI]	*p* value
Phosphorus-energy ratio	1.021 [1.012–1.031]	<0.001	1.011 [1.001–1.021]	0.030	1.015 [1.004–1.025]	0.006
PER	1.074 [1.038–1.112]	<0.001	1.037 [1.001–1.075]	0.044	1.05 [1.011–1.091]	0.011

Model 1: unadjusted. Model 2: adjusted for sex, age, diabetes and history of cardiovascular or cerebrovascular disease. Model 3: additionally adjusted for serum urea and serum albumin, with daily protein intake included for phosphorus-energy ratio models and daily phosphorus intake included for PER models, respectively.

### Association of protein-energy ratio (PER) and mortality

**Figure 4:**
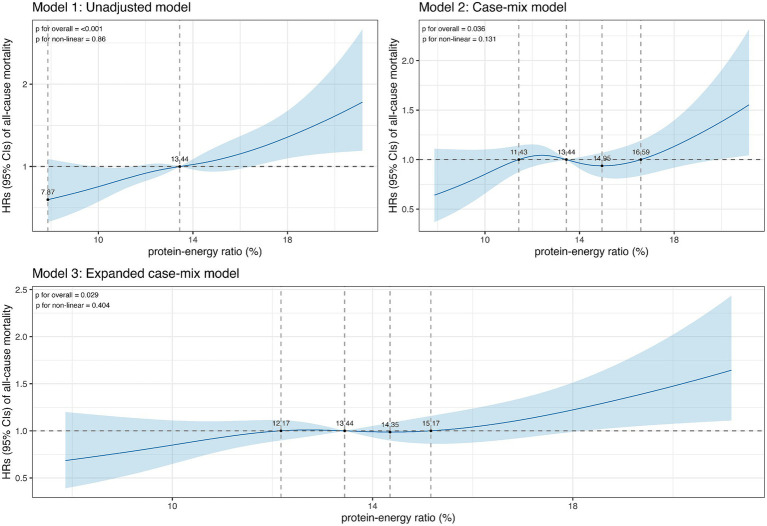
Restricted cubic spline analyses of the association between protein-energy ratio (PER) and all-cause mortality. Solid lines represent the estimated hazard ratios from the restricted cubic spline model, and shaded bands represent the 95% confidence intervals. The horizontal dashed line indicates a hazard ratio of 1.0. (Model 1: unadjusted; Model 2: adjusted for sex, age, diabetes, and history of cardiovascular or cerebrovascular disease; Model 3: additionally adjusted for serum urea, serum albumin, and daily phosphorus intake).

The four-knot RCS analyses demonstrated a linear association between PER and all-cause mortality across all three models (*p* for overall < 0.05, *p* for nonlinear > 0.05, Figure 4). No significant linear or nonlinear association was observed with cardiovascular mortality (*p* for overall > 0.05, *p* for nonlinear > 0.05, Supplementary Figure S4).

Therefore, PER was modeled as a continuous variable in Cox regression analyses. Across all models, higher PER values were associated with increased mortality risk. In the expanded model, each 1% increase in PER was associated with a 1.05-fold increase in the risk of all-cause mortality (HR = 1.050, 95% CI 1.011–1.091, *p* = 0.011; Table 3). Cox regression analyses for cardiovascular mortality showed no significant association between PER and cardiovascular death in the expanded model (Supplementary Table S4), consistent with the RCS findings.

### Sensitivity and subgroup analyses

Fine-Gray competing risk analyses for the phosphorus-protein ratio demonstrated that, compared with Q2, Q3, Q4, and Q5 were all associated with increased risks of cardiovascular mortality across all models (*p* < 0.05, Supplementary Table S5), consistent with the Cox regression results. After excluding participants who died within the first year of follow-up, the sensitivity analysis yielded results largely consistent with the primary analysis (Supplementary Tables S6, S7). However, when using the average dietary intake over the first 2 years of follow-up as the exposure, no significant associations were observed between the phosphorus-protein ratio, phosphorus-energy ratio, or PER and mortality risk in the fully adjusted models (Supplementary Tables S8, S9).

Subgroup analyses revealed a significant interaction between the phosphorus-protein ratio and history of CCVD on cardiovascular mortality (*p* for interaction = 0.020; Supplementary Figure S5). Among patients without a prior history of CCVD, all quintiles other than Q2 were associated with higher risks of cardiovascular mortality. A significant interaction was also observed between the phosphorus-energy ratio and age for all-cause mortality (*p* for interaction = 0.009; Supplementary Figure S6). Specifically, among patients older than 60 years, higher phosphorus-energy ratios were associated with an increased risk of all-cause mortality (HR = 1.250, 95% CI 1.110–1.410, *p* < 0.001).

## Discussion

Based on a retrospective PD cohort, this study is the first to simultaneously evaluate the associations of three dietary density indicators, dietary phosphorus-protein ratio, phosphorus-energy ratio, and PER, with mortality risk. The principal findings were as follows: (1) the baseline phosphorus-protein ratio exhibited a nonlinear association with both all-cause and cardiovascular mortality, with cardiovascular risk increasing markedly at moderate-to-high levels; (2) the baseline phosphorus-energy ratio and PER showed stable linear positive associations with all-cause mortality. These findings raise the possibility that baseline phosphorus-protein ratio, phosphorus-energy ratio, and PER may serve as useful prognostic markers for predicting mortality. Among patients undergoing PD, the proportional structure of nutrients within the diet, rather than the absolute intake of individual nutrients, might play a more important role in stratifying mortality risk.

In the present study, the phosphorus-protein ratio appeared to be nonlinearly associated with both all-cause and cardiovascular mortality. Additionally, in the Cox regression analysis based on baseline data, using Q2 as the reference group, all other groups showed varying degrees of increased risk of cardiovascular mortality. These findings raise the possibility that the phosphorus-protein ratio may have an “optimal range.” According to the RCS analysis in this study, the optimal range was 13–15 mg/g; both higher and lower levels were associated with adverse outcomes in patients undergoing PD. From a mechanistic perspective, as mentioned in the introduction, phosphorus is highly correlated with protein, and limiting phosphorus increases the risk of PEW, while increasing protein intake may lead to an increase in phosphorus load. In this study, moderately higher levels of phosphorus-protein ratio intake were associated with an increased risk of cardiovascular mortality among patients undergoing PD. This finding tentatively suggests that reducing phosphorus burden by selecting protein sources with relatively lower phosphorus-protein ratios may be a potentially useful dietary approach for phosphorus management in PD patients. From a dietary structural perspective, this finding is consistent with the integrated strategy advocated in current guidelines, which emphasizes controlling phosphorus burden while maintaining an appropriate level of protein intake. According to the KDIGO recommendations, patients undergoing PD should maintain a protein intake of approximately 1.0–1.2 g/kg/day, while dietary phosphorus intake should be restricted to reduce the risk of long-term complications related to Chronic Kidney Disease-Mineral and Bone Disorder (4).

KDIGO guidelines further emphasize the importance of distinguishing phosphorus sources according to protein origin. Compared with animal protein, plant-based protein sources are generally more favorable for phosphorus control, largely due to differences in phosphorus bioavailability. Specifically, the absorption rate of plant-derived phosphate is approximately 20–50%, compared with 40–60% for animal-derived phosphate, while inorganic phosphate additives are almost completely absorbed. Therefore, although certain plant-based foods such as legumes may contain relatively high phosphorus content, their lower absorption rates render them acceptable protein sources in the absence of excessive intake of other restricted nutrients (e.g., potassium) (4). Moreover, studies by Barsotti et al. (20) and Sanchis et al. (21) have shown that higher intake of plant protein was not associated with higher serum phosphorus concentrations, further supporting phosphorus management strategies based on optimization of food sources.

Our results showed that higher dietary phosphorus-energy ratio and PER were associated with increased all-cause mortality. Our findings are also in line with previous epidemiological evidence. In a cohort study of 10,030 participants, Yoon et al. (15) reported that dietary phosphorus density (i.e., phosphorus-energy ratio) was significantly associated with incident CKD among individuals with diabetes and preserved kidney function. Chang et al. (22), in a cohort of 9,686 healthy U.S. adults, reported that phosphorus density exceeding 0.35 mg/kcal was associated with an increased risk of all-cause mortality. Furthermore, Li et al. (23) analyzed 13,490 participants from the Third National Health and Nutrition Examination Survey (NHANES III), a representative cohort of the general population, and reported a positive association between PER and mortality. RCS analysis indicated that all-cause mortality risk increased markedly when PER exceeded 14.8%. Both human and animal studies in that investigation suggested that high-protein diets may impair renal hemodynamics, leading to glomerular hyperperfusion and absolute hyperfiltration, ultimately resulting in irreversible kidney damage. Although the aforementioned studies were primarily conducted in non-dialysis or general populations, evidence specifically focusing on patients undergoing PD remains limited. In patients undergoing PD, higher PER and phosphorus-energy ratio may both reflect a dietary imbalance that could be related to energy intake. In conditions of energy deficiency, the body may increase the catabolism of both endogenous and dietary protein to meet metabolic demands. Additionally, during PD, plasma proteins are lost into the dialysate due to the concentration gradient between blood and dialysate. The combination of protein loss and increased protein catabolism further exacerbates negative nitrogen balance and promotes the development of PEW (24, 25). These findings underscore the importance of ensuring adequate energy intake while controlling phosphorus and maintaining appropriate protein intake in PD patients.

In this study, when the 2-year average was used in sensitivity analyses to replace baseline values, all associations were no longer significant in the fully adjusted models. This may be explained by several factors. On the one hand, calculating a 2-year average requires patients to survive for at least 2 years, thereby excluding high-risk individuals who died early and introducing a “healthy survivor bias,” which reduces population heterogeneity and statistical power. On the other hand, changes in clinical conditions (e.g., decline in residual renal function and development of new complications) during the 2-year follow-up period may influence patients’ dietary intake.

Collectively, our findings indicate that effective dietary management in PD patients should not rely solely on reducing phosphorus intake, but rather should focus on optimizing overall dietary structure and considering phosphorus sources and bioavailability. Possible clinical strategies for managing phosphorus intake include selecting plant-based foods with lower bioavailability, choosing animal protein sources with lower phosphorus-protein ratios (e.g., lamb or tuna), and employing cooking methods such as boiling to decrease absorbable phosphorus content in high-protein foods, while simultaneously ensuring adequate energy intake.

Several limitations should be acknowledged. First, this was a single-center retrospective cohort study, and all participants were recruited from the same medical institution; therefore, dietary habits, educational level, and cultural background may limit the generalizability of our findings. Second, dietary intake was assessed using 3-day dietary records, a method widely used in clinical research but still subject to recall bias, day-to-day intake variability, and incomplete reporting, potentially resulting in measurement error. Third, although multiple covariates were adjusted, residual confounding cannot be excluded. Important factors such as inflammatory status, changes in residual renal function or dialysis adequacy, and detailed dietary characteristics (e.g., phosphorus sources) were not available in the present study.

Future studies should include multi-center prospective cohort studies or randomized controlled trials to validate the causal effects of low phosphorus-protein ratio and low phosphorus-energy ratio diets on cardiovascular and all-cause mortality in PD patients. Incorporating comprehensive nutritional quality indicators, including food sources, phosphorus bioavailability, and phosphate additive intake, may further elucidate the relationship between dietary phosphorus structure and prognosis in dialysis patients, thereby facilitating more precise nutritional interventions.

## Conclusion

Based on the findings of this study, among patients undergoing PD, a higher baseline dietary phosphorus-protein ratio appears to be associated with increased cardiovascular mortality, while higher baseline dietary phosphorus-energy ratio and PER show a dose-dependent association with increased all-cause mortality. These findings suggest that all three nutritional density indicators may serve as potential markers of dietary quality in PD patients. Specifically, the phosphorus-protein ratio might be more reflective of cardiovascular risk, while the phosphorus-energy ratio and PER appear to be more predictive of all-cause mortality. Under conditions of adequate protein intake, optimizing protein sources, reducing intake of highly bioavailable phosphorus, and improving overall dietary structure may help improve long-term outcomes in PD patients. Further studies are needed to confirm causality and to explore more precise and individualized dietary management strategies.

## Data Availability

The raw data supporting the conclusions of this article will be made available by the authors, without undue reservation.

## References

[1] United States Renal Data System (2025) Annual Data Report (ADR): United States Renal Data System. Available online at: https://usrds-adr.niddk.nih.gov/2025/end-stage-renal-disease/6-mortality (Accessed 1 January 2026).

[2] Hiruy AF Opoku S Xiong Q Jin Q Zhao J Lin X . Nutritional predictors associated with malnutrition in continuous ambulatory peritoneal dialysis patients. Clin Nutr ESPEN. (2021) 45:454–61. doi: 10.1016/j.clnesp.2021.06.033, 34620355

[3] Kiebalo T Holotka J Habura I Pawlaczyk K. Nutritional status in peritoneal Dialysis: nutritional guidelines, adequacy and the Management of Malnutrition. Nutrients. (2020) 12:1715. doi: 10.3390/nu12061715, 32521626 PMC7352713

[4] Kidney Disease: Improving Global Outcomes CKDWG. KDIGO 2024 clinical practice guideline for the evaluation and management of chronic kidney disease. Kidney Int. (2024) 105:S117–314. doi: 10.1016/j.kint.2023.10.018 38490803

[5] Cernaro V Calderone M Gembillo G Calabrese V Casuscelli C Lo Re C . Phosphate control in peritoneal Dialysis patients: issues, solutions, and open questions. Nutrients. (2023) 15:3161. doi: 10.3390/nu15143161, 37513579 PMC10386128

[6] Shammas A Joshi S Shah AD. Nutrition in peritoneal Dialysis. Adv Kidney Dis Health. (2023) 30:537–45. doi: 10.1053/j.akdh.2023.12.008, 38453271

[7] Lin W Wu J Zhan X Wen Y Wang X Feng X . A combined nutritional index and mortality in patients with peritoneal dialysis. Ren Fail. (2025) 47:2541069. doi: 10.1080/0886022X.2025.2541069, 40785303 PMC12340938

[8] Fouque D Kalantar-Zadeh K Kopple J Cano N Chauveau P Cuppari L . A proposed nomenclature and diagnostic criteria for protein-energy wasting in acute and chronic kidney disease. Kidney Int. (2008) 73:391–8. doi: 10.1038/sj.ki.5002585, 18094682

[9] Carrero JJ Thomas F Nagy K Arogundade F Avesani CM Chan M . Global prevalence of protein-energy wasting in kidney disease: a meta-analysis of contemporary observational studies from the International Society of Renal Nutrition and Metabolism. J Ren Nutr. (2018) 28:380–92. doi: 10.1053/j.jrn.2018.08.006, 30348259

[10] Carrero JJ Stenvinkel P Cuppari L Ikizler TA Kalantar-Zadeh K Kaysen G . Etiology of the protein-energy wasting syndrome in chronic kidney disease: a consensus statement from the International Society of Renal Nutrition and Metabolism (ISRNM). J Ren Nutr. (2013) 23:77–90. doi: 10.1053/j.jrn.2013.01.001, 23428357

[11] Schneider ST Klug A Andrade JM. Phosphorus knowledge and dietary intake of phosphorus of US adults undergoing Dialysis. Nutrients. (2024) 16:2034. doi: 10.3390/nu16132034, 38999782 PMC11243062

[12] Picard K Mager DR Richard C. The impact of protein type on phosphorus intake, serum phosphate concentrations, and nutrition status in adults with chronic kidney disease: a critical review. Adv Nutr. (2021) 12:2099–111. doi: 10.1093/advances/nmab062, 34113962 PMC8634523

[13] Puchulu MB Ogonowski N Sanchez-Meza F Espinosa-Cuevas MLA Miranda-Alatriste P. Dietary phosphorus to protein ratio for the Mexican population with chronic kidney disease. J Am Coll Nutr. (2019) 38:247–58. doi: 10.1080/07315724.2018.1501327, 30257134

[14] Gwin JA Karl JP Lutz LJ Gaffney-Stomberg E McClung JP Pasiakos SM. Higher protein density diets are associated with greater diet quality and micronutrient intake in healthy young adults. Front Nutr. (2019) 6:59. doi: 10.3389/fnut.2019.00059, 31134205 PMC6514148

[15] Yoon CY Park JT Jhee JH Noh J Kee YK Seo C . High dietary phosphorus density is a risk factor for incident chronic kidney disease development in diabetic subjects: a community-based prospective cohort study. Am J Clin Nutr. (2017) 106:311–21. doi: 10.3945/ajcn.116.151654, 28592606

[16] Yang Y Wang G Pan X. China Food Composition Table. Beijing: Peking University Medical Press (2009).

[17] FAO/WHO/UNU. Energy and protein requirements. Report of a Joint FAO/WHO/UNU Expert Consultation. Geneva: World Health Organization (1985). 3937340

[18] Desquilbet L Mariotti F. Dose-response analyses using restricted cubic spline functions in public health research. Stat Med. (2010) 29:1037–57. doi: 10.1002/sim.3841, 20087875

[19] Frank E Harrell J. Regression Modeling Strategies: With Applications to linear Models, Logistic and Ordinal Regression, and Survival Analysis. 2nd ed. Cham: Springer (2015).

[20] Barsotti G Morelli E Cupisti A Meola M Dani L Giovannetti S. A low-nitrogen low-phosphorus vegan diet for patients with chronic renal failure. Nephron. (1996) 74:390–4. doi: 10.1159/000189341, 8893161

[21] Sanchis P Molina M Berga F Munoz E Fortuny R Costa-Bauza A . A pilot randomized crossover trial assessing the safety and short-term effects of walnut consumption by patients with chronic kidney disease. Nutrients. (2019) 12:31881702. doi: 10.3390/nu12010063 PMC7020056 31881702

[22] Chang AR Lazo M Appel LJ Gutierrez OM Grams ME. High dietary phosphorus intake is associated with all-cause mortality: results from NHANES III. Am J Clin Nutr. (2014) 99:320–7. doi: 10.3945/ajcn.113.073148, 24225358 PMC3893724

[23] Li Z Huang L Zhu Q Wang F Li D Qu B . Dietary total, animal, and plant protein-energy ratio and risk of mortality: results from the NHANES III and a lifelong animal experiment. Food Funct. (2025) 16:6532–46. doi: 10.1039/D4FO05785F, 40699992

[24] Mafrici B Viramontes-Horner D Gardner DS Smith K Taal MW Selby NM . Amino acid and protein losses in adult patients receiving maintenance dialysis: a literature review. J Ren Nutr. (2026) 36:30–48. doi: 10.1053/j.jrn.2025.07.012, 40759352

[25] Ito Y Ryuzaki M Sugiyama H Tomo T Yamashita AC Ishikawa Y . Peritoneal dialysis guidelines 2019 part 1 (position paper of the Japanese Society for Dialysis Therapy). Renal Replace Ther. (2021) 7:40. doi: 10.1186/s41100-021-00348-6

