# Supplementary material for: Associations of dietary phosphorus-protein ratio, phosphorus-energy ratio, and protein-energy ratio with mortality in peritoneal dialysis patients: a retrospective cohort study
Source: Front Nutr. 2026 Jun 9;13:1798804. doi: 10.3389/fnut.2026.1798804 (PMC13286775; doi:10.3389/fnut.2026.1798804)
Supplement: Supplementary file 1 [file Table_1.docx]

# Supplementary Materials

**Table S1** Proportional hazards assumption test results for phosphorus-protein ratio, phosphorus-energy ratio, and protein-energy ratio(PER) in the fully adjusted Cox model

|  |  | All-cause mortality | | |  | Cardiovascular mortality | | |
| --- | --- | --- | --- | --- | --- | --- | --- | --- |
|  |  | χ² | df | *p* value |  | χ² | df | *p* value |
| phosphorus-protein ratio | phosphorus-protein ratio | 2.105 | 4 | 0.716 |  | 3.881 | 4 | 0.422 |
|  | age | 2.248 | 1 | 0.134 |  | 0.003 | 1 | 0.956 |
|  | sex | 0.027 | 1 | 0.871 |  | 0.475 | 1 | 0.491 |
|  | diabetes | 0.004 | 1 | 0.947 |  | 1.972 | 1 | 0.160 |
|  | history of CCVD | 0.092 | 1 | 0.761 |  | 0.401 | 1 | 0.527 |
|  | serum urea | 0.175 | 1 | 0.675 |  | 1.128 | 1 | 0.288 |
|  | serum albumin | 2.950 | 1 | 0.086 |  | 3.146 | 1 | 0.076 |
|  | DEI | 0.289 | 1 | 0.591 |  | 0.179 | 1 | 0.673 |
|  | GLOBAL | 9.706 | 11 | 0.557 |  | 10.404 | 11 | 0.494 |
|  |  |  |  |  |  |  |  |  |
| phosphorus-energy ratio | phosphorus-energy ratio | 2.790 | 1 | 0.095 |  | 0.013 | 1 | 0.910 |
|  | sex | 0.008 | 1 | 0.930 |  | 0.448 | 1 | 0.503 |
|  | age | 1.692 | 1 | 0.193 |  | 0.000 | 1 | 0.991 |
|  | diabetes | 0.002 | 1 | 0.960 |  | 1.962 | 1 | 0.161 |
|  | history of CCVD | 0.139 | 1 | 0.709 |  | 0.380 | 1 | 0.538 |
|  | serum urea | 0.073 | 1 | 0.788 |  | 1.128 | 1 | 0.288 |
|  | serum albumin | 3.472 | 1 | 0.062 |  | 2.973 | 1 | 0.085 |
|  | protein intake | 0.088 | 1 | 0.767 |  | 0.852 | 1 | 0.356 |
|  | GLOBAL | 9.841 | 8 | 0.276 |  | 6.714 | 8 | 0.568 |
|  |  |  |  |  |  |  |  |  |
| PER | PER | 1.410 | 1 | 0.235 |  | 0.311 | 1 | 0.577 |
|  | sex | 0.006 | 1 | 0.938 |  | 0.535 | 1 | 0.464 |
|  | age | 1.639 | 1 | 0.200 |  | 0.024 | 1 | 0.876 |
|  | diabetes | 0.026 | 1 | 0.873 |  | 1.825 | 1 | 0.177 |
|  | history of CCVD | 0.163 | 1 | 0.686 |  | 0.283 | 1 | 0.595 |
|  | serum urea | 0.137 | 1 | 0.711 |  | 1.074 | 1 | 0.300 |
|  | serum albumin | 3.204 | 1 | 0.073 |  | 3.014 | 1 | 0.083 |
|  | phosphorus intake | 0.593 | 1 | 0.441 |  | 0.212 | 1 | 0.645 |
|  | GLOBAL | 7.706 | 8 | 0.463 |  | 6.779 | 8 | 0.561 |

DEI: dietary energy intake;CCVD:cardiovascular or cerebrovascular disease;PER:protein-energy ratio

**Table S2** Univariate Cox regression analyses of all-cause and cardiovascular mortality

|  | All-cause mortality | | Cardiovascular mortality | |
| --- | --- | --- | --- | --- |
|  | HR[95%CI] | *p* value | HR[95%CI] | *p* value |
| sex | 1.012[0.834-1.229] | 0.901 | 1.309[0.954-1.795] | 0.095 |
| diabetes | 1.721[1.416-2.093] | **<0.001** | 2.358[1.713-3.246] | **<0.001** |
| hypertension | 1.150[0.829-1.595] | 0.403 | 1.159[0.680-1.975] | 0.587 |
| history of CCVD | 2.373[1.941-2.901] | **<0.001** | 3.227[2.344-4.442] | **<0.001** |
| age | 1.062[1.053-1.071] | **<0.001** | 1.046[1.033-1.059] | **<0.001** |
| BMI | 0.984[0.958-1.010] | 0.225 | 1.012[0.971-1.055] | 0.567 |
| Kt/V | 1.060[0.898-1.251] | 0.491 | 0.997[0.759-1.309] | 0.982 |
| GFR | 1.007[0.966-1.049] | 0.757 | 1.070[1.005-1.139] | **0.034** |
| serum urea | 0.952[0.935-0.970] | **<0.001** | 0.946[0.918-0.975] | **<0.001** |
| serum creatinine | 0.844[0.811-0.878] | **<0.001** | 0.828[0.775-0.885] | **<0.001** |
| serum albumin | 0.913[0.895-0.931] | **<0.001** | 0.819[0.543-1.235] | 0.341 |
| serum phosphorus | 0.798[0.618-1.030] | 0.084 | 0.922[0.893-0.953] | **<0.001** |
| daily protein intake | 0.992[0.985-0.999] | **0.026** | 0.994[0.983-1.005] | 0.284 |
| nDPI | 0.755[0.497-1.147] | 0.188 | 0.720[0.365-1.417] | 0.341 |
| DEI | 0.999[0.999-1.000] | **<0.001** | 1.000[0.999-1.000] | 0.125 |
| nDEI | 0.966[0.951-0.981] | **<0.001** | 0.979[0.955-1.003] | 0.091 |
| daily phosphorus intake | 0.620[0.386-0.997] | **0.049** | 0.954[0.453-2.012] | 0.902 |
| phosphorus-protein ratio | 1.014[0.965-1.065] | 0.585 | 1.079[0.998-1.167] | 0.055 |
| phosphorus-energy ratio | 1.021[1.011-1.030] | **<0.001** | 1.017[1.001,1.032] | **0.032** |
| PER | 1.074[1.038-1.112] | **<0.001** | 1.025[0.966-1.087] | 0.412 |

BMI: body mass index; CCVD: cardiovascular or cerebrovascular disease; GFR: glomerular filtration rate; nDPI: normalized dietary protein intake; DEI: dietary energy intake; nDEI: normalized dietary energy intake; PER:protein-energy ratio

**Table S3** Model fit statistics for restricted cubic spline models of the phosphorus-protein ratio using different numbers of knots

| Number | Model | Knots | AIC(Cardiovascular mortality) | AIC(All-cause mortality) |
| --- | --- | --- | --- | --- |
| 1 | Model 1 | 3 | 1811.74 | 4763.965 |
| 2 | Model 1 | 4 | 1812.875 | 4764.449 |
| 3 | Model 1 | 5 | 1814.198 | 4766.685 |
| 4 | Model 1 | 6 | 1812.398 | 4763.686 |
| 5 | Model 1 | 7 | 1808.967 | 4763.468 |
| 6 | Model 1 | 8 | 1811.660 | 4765.639 |
| 7 | Model 2 | 3 | 1723.103 | 4508.475 |
| 8 | Model 2 | 4 | 1724.230 | 4510.126 |
| 9 | Model 2 | 5 | 1726.346 | 4508.988 |
| 10 | Model 2 | 6 | 1722.894 | 4504.610 |
| 11 | Model 2 | 7 | 1722.059 | 4505.360 |
| 12 | Model 2 | 8 | 1724.181 | 4507.101 |
| 13 | Model 3 | 3 | 1715.638 | 4470.207 |
| 14 | Model 3 | 4 | 1716.803 | 4472.030 |
| 15 | Model 3 | 5 | 1718.958 | 4472.267 |
| 16 | Model 3 | 6 | 1714.997 | 4466.709 |
| 17 | Model 3 | 7 | 1713.263 | 4466.088 |
| 18 | Model 3 | 8 | 1715.586 | 4467.659 |

Model 1: unadjusted. Model 2: adjusted for sex, age, diabetes and history of cardiovascular or cerebrovascular disease. Model 3: additionally adjusted for serum urea, serum albumin and daily energy intake

**Table S4** Associations of phosphorus-energy ratio and protein-energy ratio (PER) with cardiovascular mortality

|  | Model1 | Model2 | Model3 |
| --- | --- | --- | --- |
| phosphorus-energy ratio |  |  |  |
| HR[95%CI] | 1.017[1.001-1.032] | 1.007[0.991-1.023] | 1.009[0.993-1.026] |
| *p* value | 0.033 | 0.394 | 0.27 |
| PER |  |  |  |
| HR[95%CI] | 1.025[0.966-1.087] | 0.992[0.936-1.05] | 0.991[0.93-1.056] |
| *p* value | 0.412 | 0.774 | 0.781 |

Model 1: unadjusted. Model 2: adjusted for sex, age, diabetes and history of cardiovascular or cerebrovascular disease. Model 3: additionally adjusted for serum urea and serum albumin, with daily protein intake included for phosphorus-energy ratio models and daily phosphorus intake included for PER models, respectively

**Table S5** Association between the phosphorus-protein ratio and cardiovascular mortality using Fine-Gray competing risk models

| **Group** | **Median (IQR)** | **Model1** | | **Model2** | | **Model3** | |
| --- | --- | --- | --- | --- | --- | --- | --- |
|  |  | HR[95%CI] | *p* value | HR[95%CI] | *p* value | HR[95%CI] | *p* value |
| Q1 | 12.95[12.14,13.46] | 1.697 [0.984-2.928] | 0.057 | 1.706 [0.983-2.963] | 0.058 | 1.750 [1.006-3.044] | 0.048 |
| Q2 | 14.41[14.15,14.62] | reference | | reference | | reference | |
| Q3 | 15.30[15.10,15.50] | 2.056 [1.213-3.486] | 0.007 | 2.042 [1.193-3.495] | 0.009 | 2.117 [1.232-3.637] | 0.007 |
| Q4 | 16.21[15.91,16.58] | 1.814 [1.049-3.139] | 0.033 | 1.851 [1.064-3.222] | 0.029 | 1.870 [1.070-3.268] | 0.028 |
| Q5 | 17.91[17.38,18.62] | 2.024 [1.172-3.495] | 0.011 | 1.894 [1.086-3.304] | 0.025 | 1.925 [1.097-3.376] | 0.022 |

Model 1: unadjusted. Model 2: adjusted for sex, age, diabetes and history of cardiovascular or cerebrovascular disease. Model 3: additionally adjusted for serum urea, serum albumin and daily energy intake

**Table S6** Sensitivity analysis of phosphorus-protein ratio and all-cause and cardiovascular mortality after excluding patients who died within the first year of follow-up(n=758)

|  | Median [IQR] | Model1 | | Model2 | | Model3 | |
| --- | --- | --- | --- | --- | --- | --- | --- |
|  |  | HR[95%CI] | *p* value | HR[95%CI] | *p* value | HR[95%CI] | *p* value |
| Cardiovascular mortality |  |  |  |  |  |  |  |
| Q1 | 12.70[12.14, 13.47] | 1.188 [0.865-1.63] | 0.287 | 1.142 [0.831-1.569] | 0.414 | 1.191 [0.866-1.637] | 0.283 |
| Q2 | 14.39 [14.16, 14.62] | reference | | reference | | reference | |
| Q3 | 15.29 [15.09, 15.49] | 2.195 [1.243-3.876] | 0.007 | 2.059 [1.163-3.644] | 0.013 | 2.266 [1.277-4.02] | 0.005 |
| Q4 | 16.27 [15.91, 16.58] | 2.221 [1.246-3.958] | 0.007 | 2.394 [1.335-4.293] | 0.003 | 2.351 [1.309-4.223] | 0.004 |
| Q5 | 18.35 [17.43, 18.77] | 2.091 [1.173-3.727] | 0.012 | 1.762 [0.985-3.154] | 0.056 | 1.727 [0.962-3.097] | 0.067 |
| All-cause mortality |  |  |  |  |  |  |  |
| Q1 | 12.70 [12.14, 13.47] | 0.777 [0.667-0.907] | 0.001 | 1.009 [0.864-1.178] | 0.91 | 1.022 [0.875-1.193] | 0.786 |
| Q2 | 14.39 [14.16, 14.62] | reference | | reference | | reference | |
| Q3 | 15.29 [15.09, 15.49] | 1.264 [0.919-1.739] | 0.149 | 1.197 [0.869-1.65] | 0.271 | 1.292 [0.937-1.78] | 0.118 |
| Q4 | 16.27 [15.91, 16.58] | 1.477 [1.079-2.024] | 0.015 | 1.418 [1.033-1.947] | 0.031 | 1.387 [1.01-1.905] | 0.044 |
| Q5 | 18.35 [17.43, 18.77] | 1.101 [0.789-1.536] | 0.572 | 0.941 [0.672-1.318] | 0.724 | 0.932 [0.665-1.308] | 0.685 |

Model 1: unadjusted. Model 2: adjusted for sex, age, diabetes and history of cardiovascular or cerebrovascular disease. Model 3: additionally adjusted for serum urea, serum albumin and daily energy intake

**Table S7** Sensitivity analysis of phosphorus-energy ratio, and protein-energy ratio(PER) and all-cause and cardiovascular mortality after excluding patients who died within the first year of follow-up(n=758)

|  |  | Model1 | | Model2 | | Model3 | |
| --- | --- | --- | --- | --- | --- | --- | --- |
|  |  | HR[95%CI] | *p* value | HR[95%CI] | *p* value | HR[95%CI] | *p* value |
| All-cause mortality | phosphorus-energy ratio | 1.021 [1.011-1.031] | <0.001 | 1.011 [1.001-1.022] | 0.03 | 1.015 [1.004-1.026] | 0.007 |
|  | PER | 1.342 [1.16-1.551] | <0.001 | 1.164 [1.003-1.351] | 0.045 | 1.237 [1.057-1.448] | 0.008 |
| Cardiovascular mortality | phosphorus-energy ratio | 1.019 [1.003-1.035] | 0.023 | 1.009 [0.993-1.026] | 0.275 | 1.012 [0.994-1.029] | 0.186 |
|  | PER | 1.109 [0.867-1.418] | 0.41 | 0.97 [0.763-1.233] | 0.805 | 0.969 [0.743-1.263] | 0.814 |

Model 1: unadjusted.Model 2: adjusted for sex, age, diabetes and history of cardiovascular or cerebrovascular disease. Model 3: additionally adjusted for serum urea and serum albumin, with daily protein intake included for phosphorus–energy ratio models and daily phosphorus intake included for PER models, respectively

**Table S8** Associations of 2-year averaged phosphorus-energy ratio and protein-energy ratio(PER) with all-cause mortality: Cox proportional hazards models(n=692)

|  | Model1 | Model2 | Model3 |
| --- | --- | --- | --- |
| phosphorus-energy ratio |  |  |  |
| HR[95%CI] | 1.032[1.016-1.049] | 1.000[0.983-1.017] | 1.003[0.985-1.021] |
| *p* value | <0.001 | 0.981 | 0.759 |
| PER |  |  |  |
| HR[95%CI] | 1.564[1.213-2.016] | 1.079[0.819-1.423] | 1.191[0.888-1.596] |
| *p* value | <0.001 | 0.588 | 0.243 |

Model 1: unadjusted.Model 2: adjusted for sex, age, diabetes and history of cardiovascular or cerebrovascular disease. Model 3: additionally adjusted for serum urea and serum albumin, with daily protein intake included for phosphorus–energy ratio models and daily phosphorus intake included for PER models, respectively

**Table S9** Associations of 2-year averaged phosphorus-protein ratio with all-cause and cardiovascular mortality: Cox proportional hazards models(n=692)

|  | Median [IQR] | Model1 | | Model2 | | Model3 | |
| --- | --- | --- | --- | --- | --- | --- | --- |
|  |  | HR[95%CI] | *p* value | HR[95%CI] | *p* value | HR[95%CI] | *p* value |
| Cardiovascular mortality |  |  |  |  |  |  |  |
| Q1 | 13.44[12.84, 13.74] | 1.353[0.711-2.578] | 0.357 | 1.358[0.71-2.598] | 0.355 | 1.386[0.724-2.653] | 0.324 |
| Q2 | 14.50[14.31, 14.68] | reference | | reference | | reference | |
| Q3 | 15.25[15.09, 15.40] | 1.472[0.793-2.733] | 0.221 | 1.232[0.661-2.298] | 0.511 | 1.235[0.659-2.311] | 0.510 |
| Q4 | 15.93[15.76, 16.11] | 2.112[1.159-3.850] | 0.015 | 1.720[0.937-3.157] | 0.080 | 1.653[0.899-3.038] | 0.106 |
| Q5 | 17.14[16.63, 17.76] | 1.688[0.905-3.149] | 0.100 | 1.638[0.871-3.08] | 0.126 | 1.588[0.843-2.993] | 0.153 |
| All-cause mortality |  |  |  |  |  |  |  |
| Q1 | 13.44[12.84, 13.74] | 0.846[0.592-1.208] | 0.357 | 0.819[0.571-1.177] | 0.281 | 0.859[0.599-1.233] | 0.410 |
| Q2 | 14.50[14.31, 14.68] | reference | | reference | | reference | |
| Q3 | 15.25[15.09, 15.40] | 0.979[0.700-1.368] | 0.899 | 0.807[0.576-1.131] | 0.214 | 0.855[0.608-1.201] | 0.365 |
| Q4 | 15.93[15.76, 16.11] | 1.034[0.732-1.462] | 0.849 | 0.836[0.588-1.188] | 0.317 | 0.802[0.563-1.142] | 0.221 |
| Q5 | 17.14[16.63, 17.76] | 0.870[0.606-1.247] | 0.447 | 0.747[0.518-1.076] | 0.117 | 0.750[0.520-1.082] | 0.124 |

Model 1: unadjusted. Model 2: adjusted for sex, age, diabetes and history of cardiovascular or cerebrovascular disease. Model 3: additionally adjusted for serum urea, serum albumin and daily energy intake

**Table S10** Correlation among phosphorus-protein ratio, phosphorus-energy ratio, and protein-energy ratio(PER)

|  | **protein-energy ratio** | **phosphorus-protein ratio** | **PER** |
| --- | --- | --- | --- |
| **protein-energy ratio** | - |  |  |
| **phosphorus-protein ratio** | -0.336*** | - |  |
| **PER** | 0.791*** | 0.292*** | - |

***p<0.001


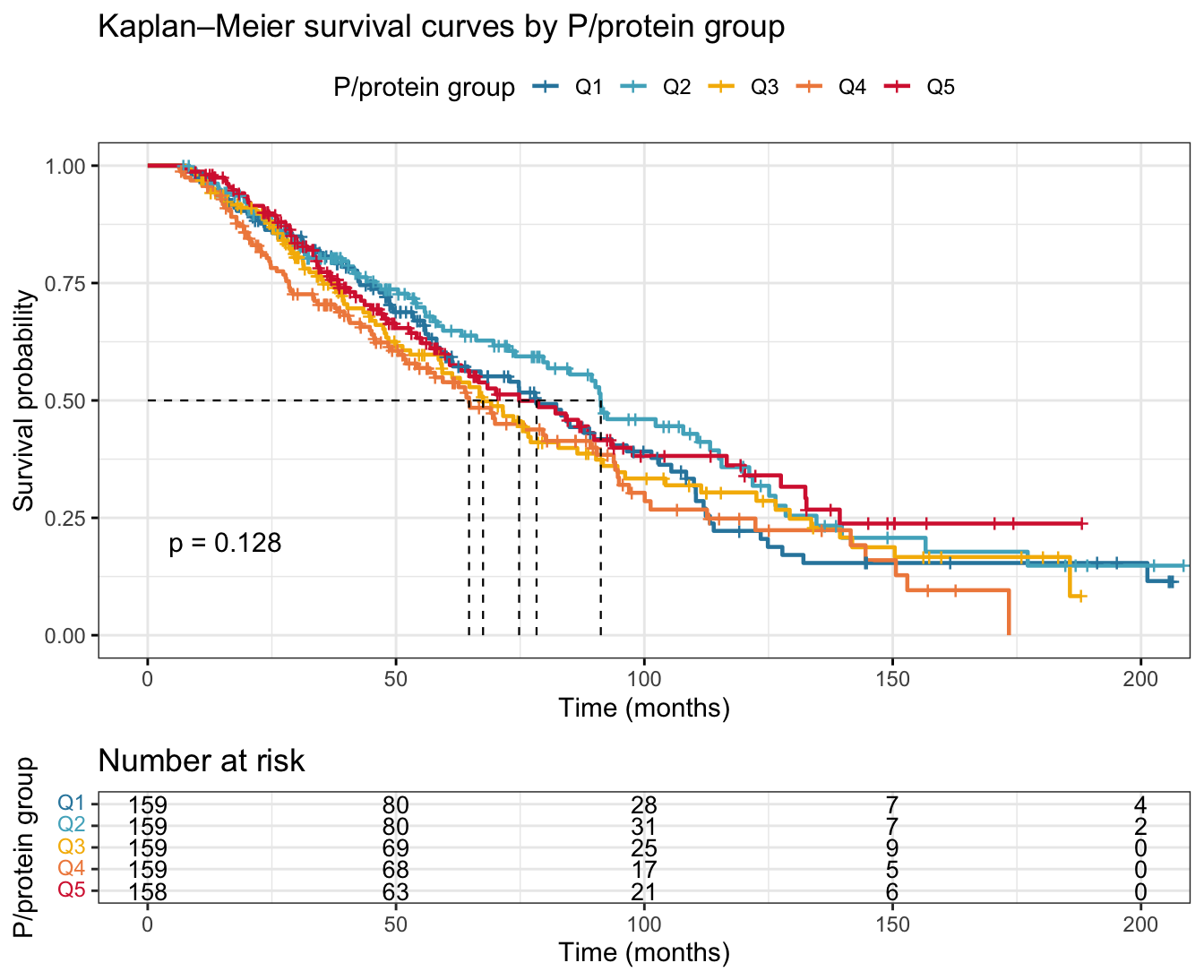


**Figure S1** Kaplan-Meier survival curves for all-cause mortality by phosphorus-protein ratio


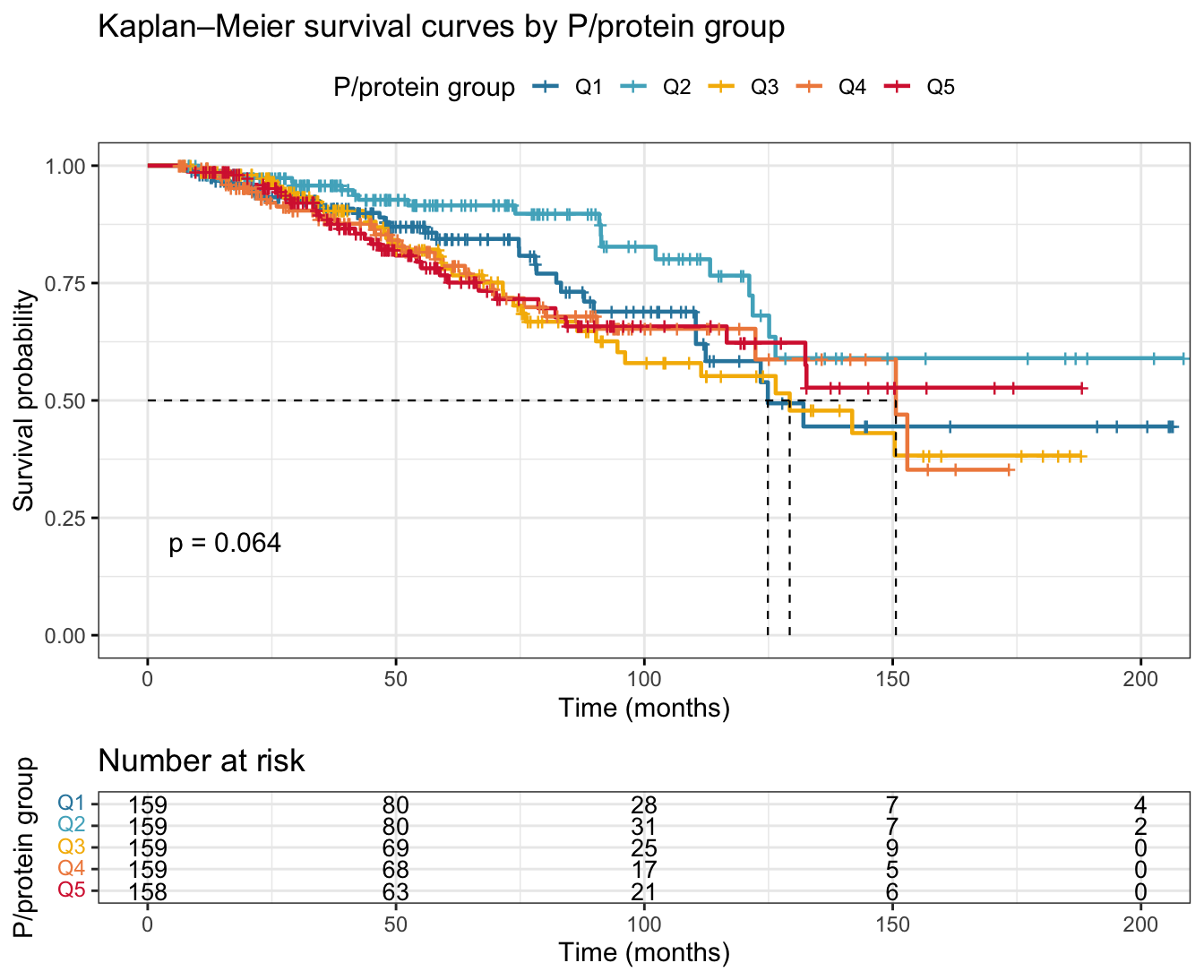


**Figure S2** Kaplan-Meier survival curves for cardiovascular mortality by phosphorus-protein ratio


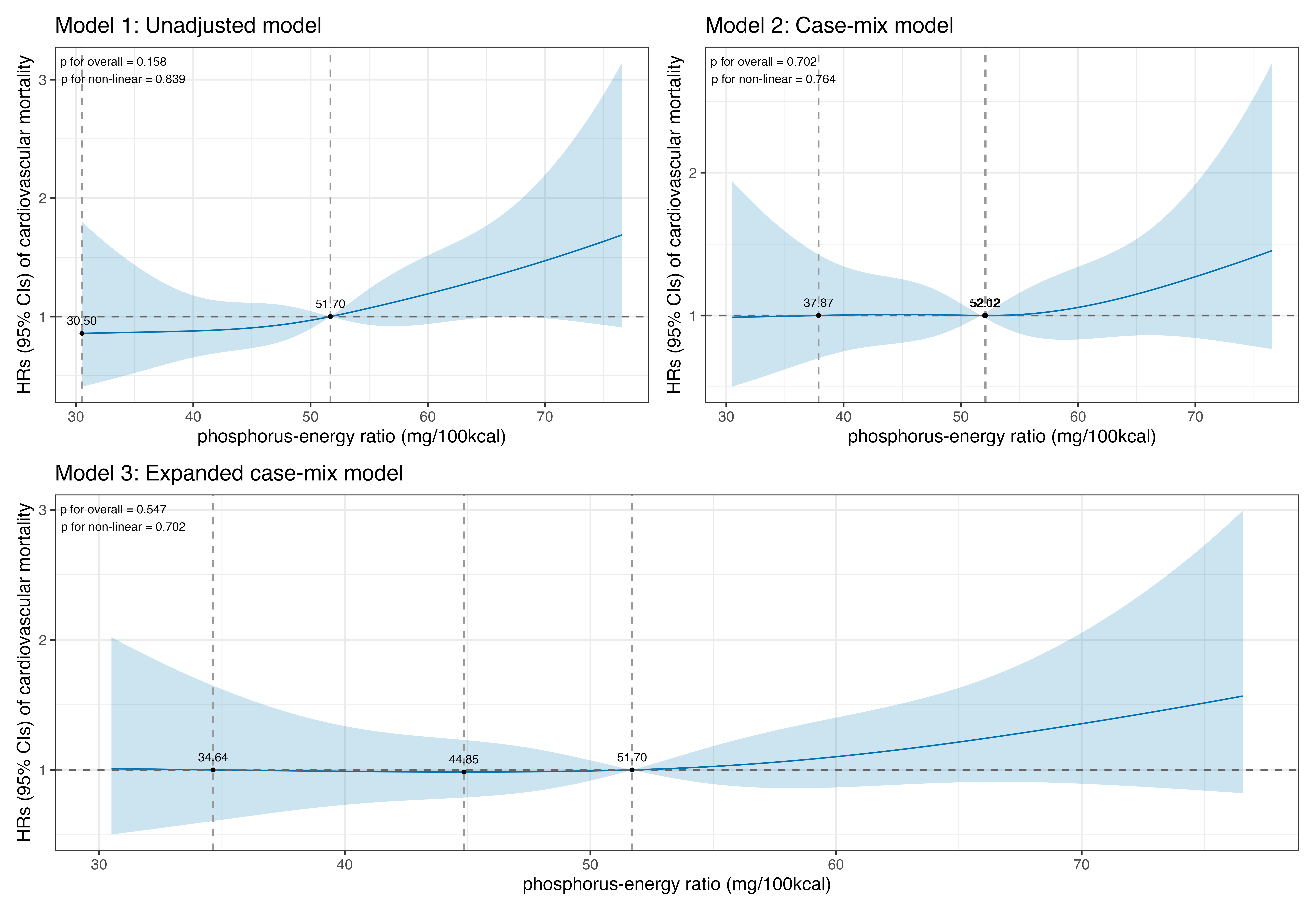


**Figure S3** Restricted cubic spline analyses of the association between phosphorus-energy ratio and cardiovascular mortality. Solid lines represent the estimated hazard ratios from the restricted cubic spline model, and shaded bands represent the 95% confidence intervals. The horizontal dashed line indicates a hazard ratio of 1.0. (Model 1: unadjusted. Model 2: adjusted for sex, age, diabetes and history of cardiovascular or cerebrovascular disease. Model 3: additionally adjusted for serum urea, serum albumin and daily protein intake.)


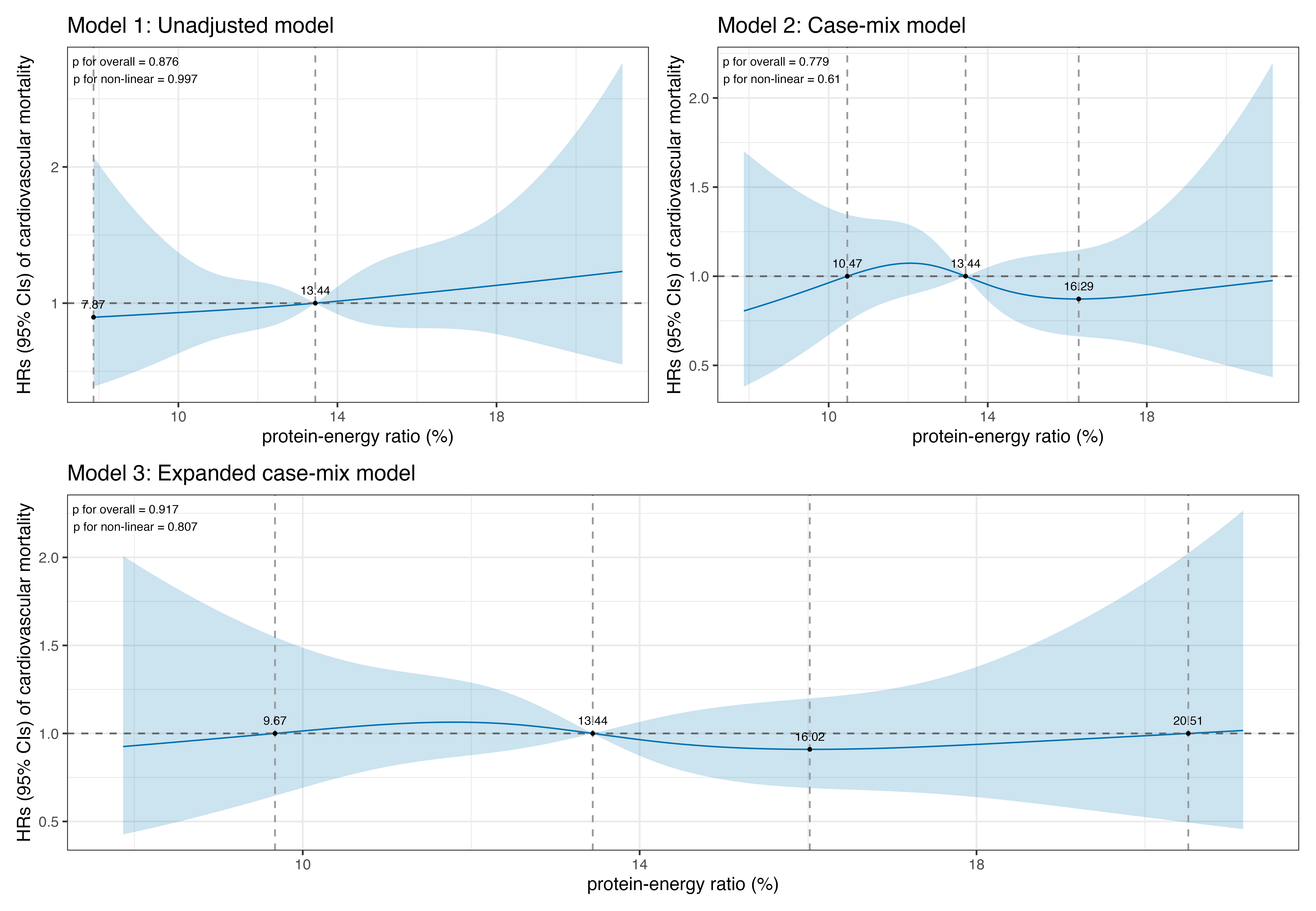


**Figure S4** Restricted cubic spline analyses of the association between protein-energy ratio(PER) and cardiovascular mortality. Solid lines represent the estimated hazard ratios from the restricted cubic spline model, and shaded bands represent the 95% confidence intervals. The horizontal dashed line indicates a hazard ratio of 1.0. (Model 1: unadjusted.Model 2: adjusted for sex, age, diabetes and history of cardiovascular or cerebrovascular disease. Model 3: additionally adjusted for serum urea,serum albumin and daily phosphorus intake.)


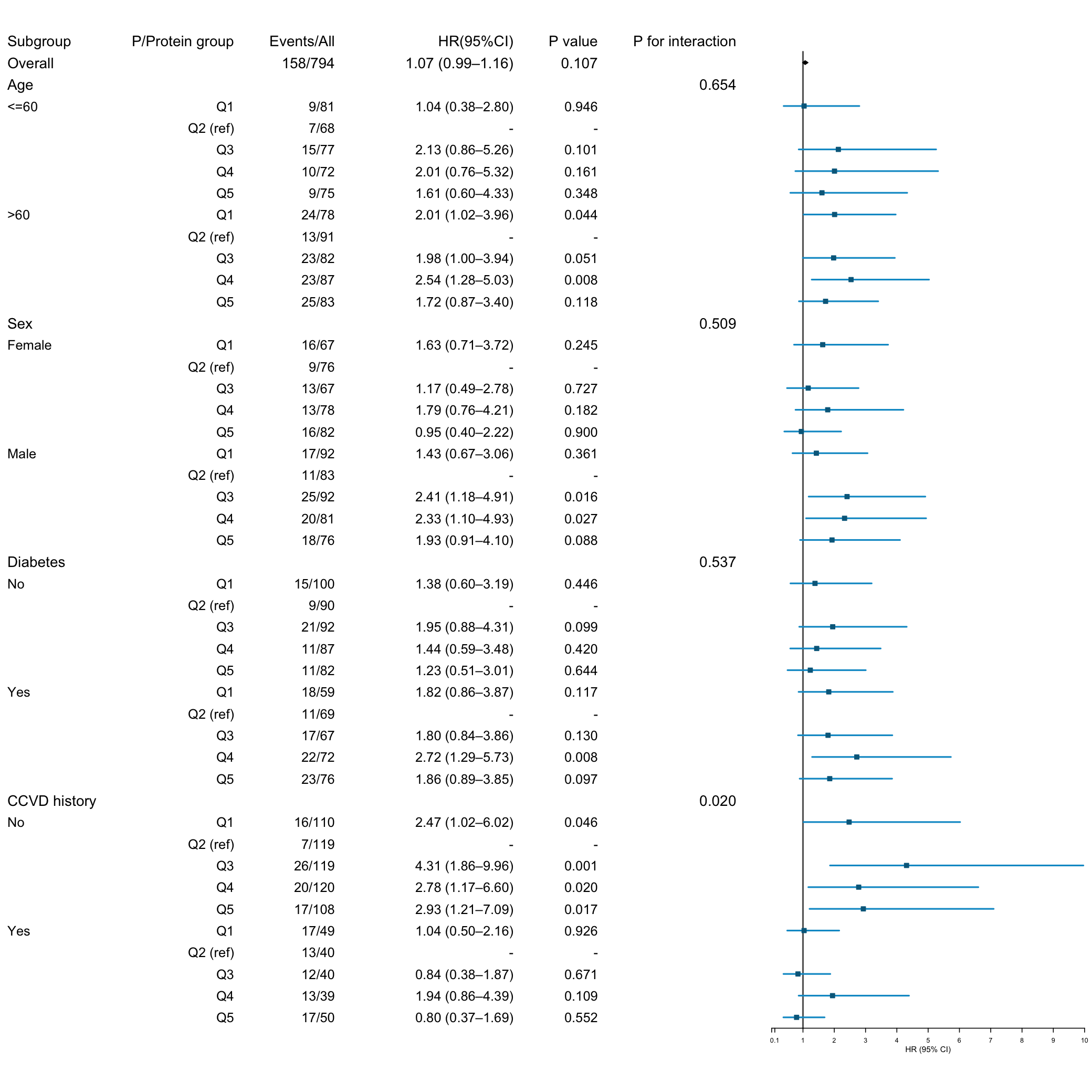


**Figure S5** Subgroup analyses of the association between phosphorus-protein ratio and cardiovascular mortality


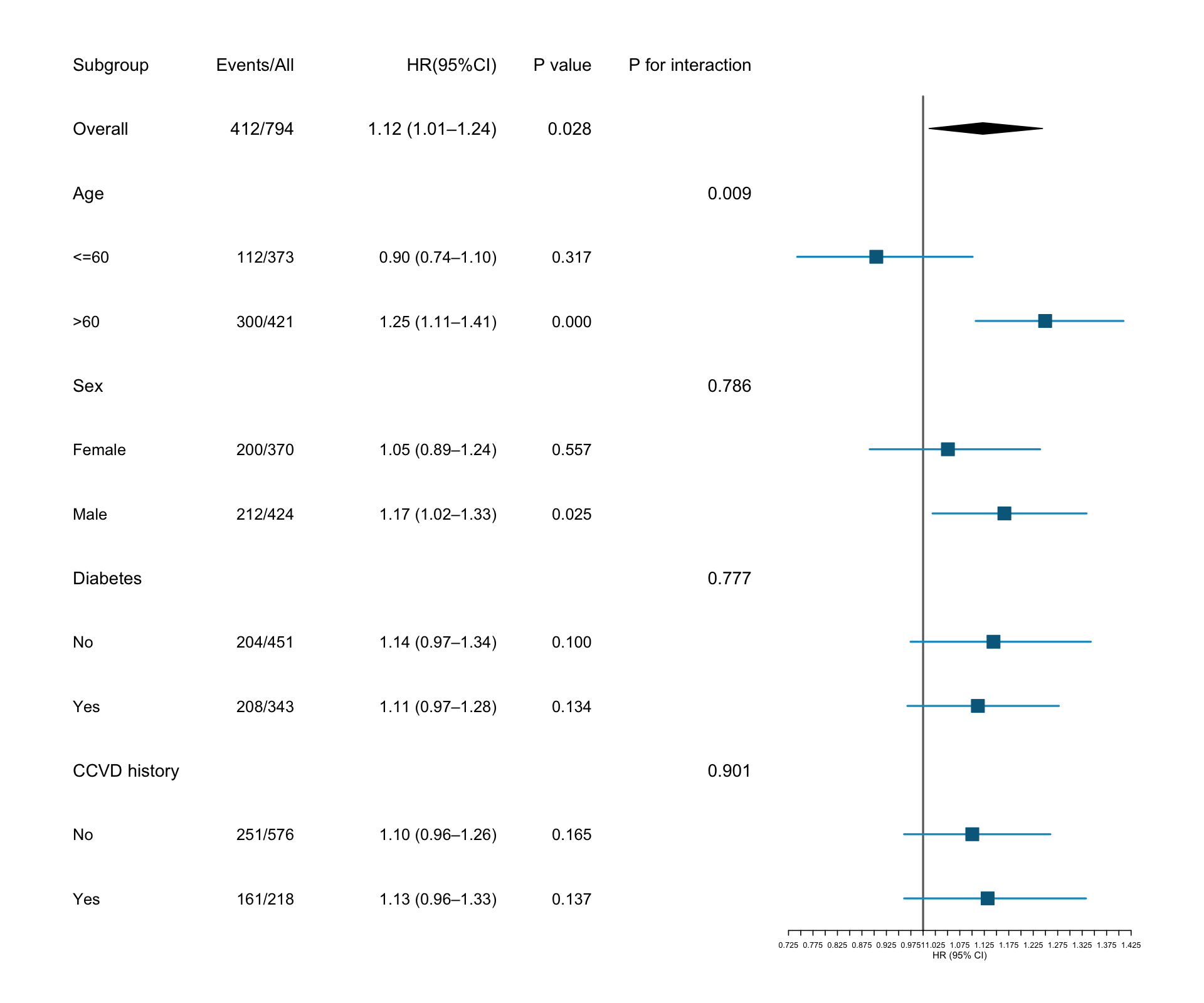


**Figure S6** Subgroup analyses of the association between phosphorus-energy ratio and all-cause mortality


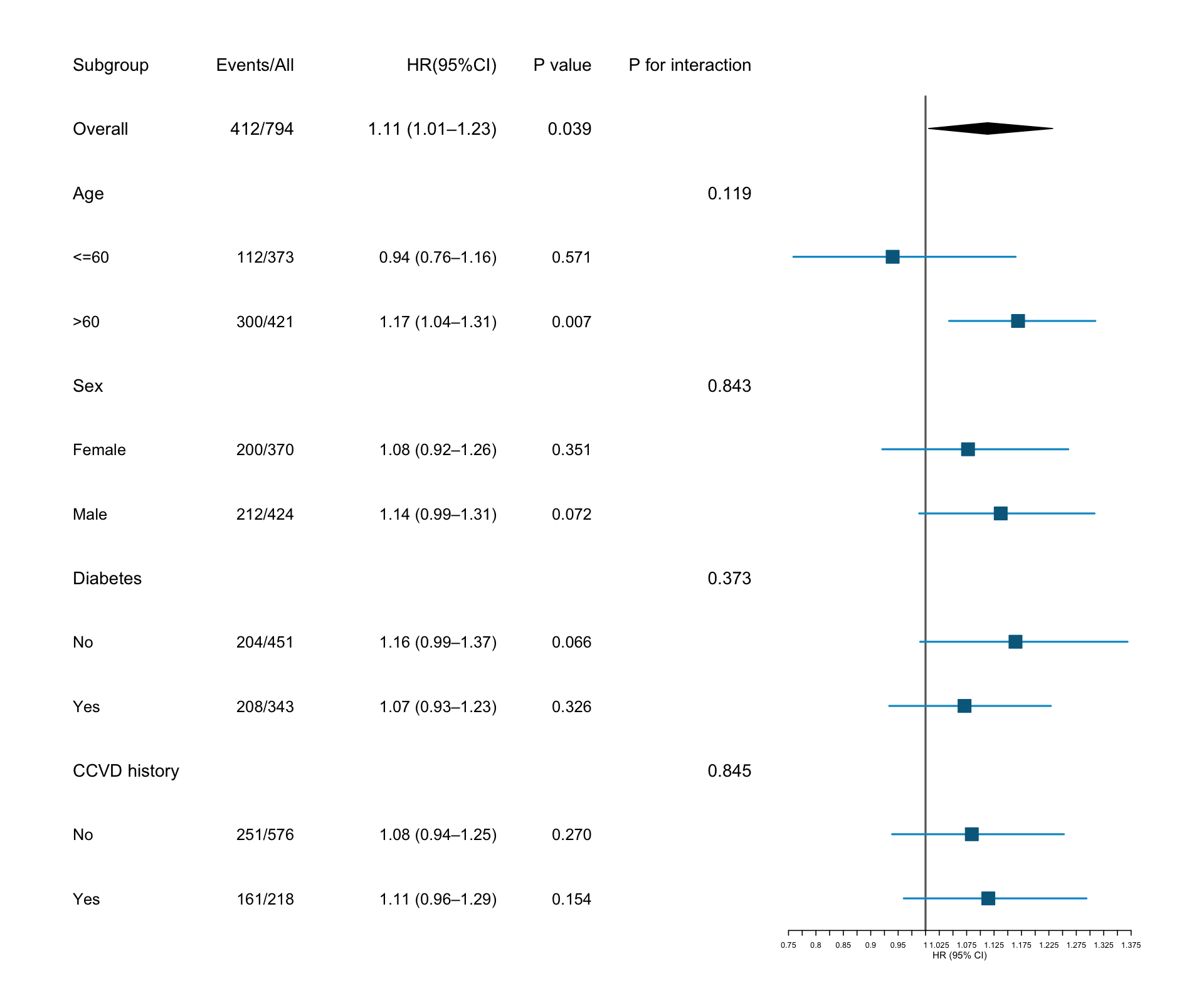


**Figure S7** Subgroup analyses of the association between protein-energy ratio(PER) and all-cause mortality
